# MMPI analysis of 12345 convenience hotline operators

**DOI:** 10.3389/fpsyt.2025.1644345

**Published:** 2025-10-20

**Authors:** Lin Ma, Yongsheng Tong, Tongtong Wang, Dong Zhang, Xingjie Yang, Haiting Xu, Xiao Liu

**Affiliations:** Beijing Huilongguan Hospital, Beijing, China

**Keywords:** 12345 Operator, convenience hotline, psychological characteristics, occupational stress, MMPI

## Abstract

**Objective:**

This study aimed to assess 12345 Government Service and Convenience Hotline operators using the Minnesota Multiphasic Personality Inventory (MMPI), with the goal of identifying their personality traits and psychological characteristics. Drawing on these findings, constructive recommendations were proposed to improve the operators’ mental well-being, thereby enhancing the overall quality of their service provision.

**Methods:**

A total of 971 operators from the government service and convenience hotline in City B were recruited as participants. The Minnesota Multiphasic Personality Inventory (MMPI) was used to assess the participants’ psychological characteristics.

**Results:**

The overall rate of abnormal psychological characteristics was 76.1%, with 28.53% showing mild abnormalities, 11.12% moderate abnormalities, and 36.46% severe abnormalities. Repression was the only subscale with the mean score exceeded the borderline normal threshold (T >60), and fewer than 50% of participants scored within the borderline normal range. There were significant differences in five aspects of hypomania: Hypomania, Correction, Dependence, Social Responsibility, and Bias between operators with moderately and severely abnormal mental status and those with normally and mildly abnormal mental status (*p* < 0.001). The results of multiple subscales of male operators were significantly different from those of female operators (*p* < 0.05).

**Conclusion:**

The mental status of 12345 public service hotline operators was found to be suboptimal, with high prevalence of occupational burnout. Operators with moderate - to - severe abnormal mental status faced limited psychological resources, which hindered their professional performance. This study also identified gender-based differences in the mental status of 12345 hotline operators. Male and female hotline operators each possess distinct strengths in hotline service delivery. Training supervisors to recognize the early signs of burnout is not only important but also meaningful, as it helps ensure the smooth operation of 12345 Government Service Hotline services.

## 1 Introduction

12345 Government Service and Convenience Hotline (12345 Hotline for short) refers to the public service platform set up by local municipal people’s governments, which is composed of telephone 12345, mayor’s mailbox, mobile phone SMS, mobile phone client, Weibo, WeChat, and other means specially designed to serve as a citizen service hotline. The core responsibility of operators at the 12345 Hotline is to serve as a communication bridge between the government, the public, and enterprises. By answering incoming calls, they address a wide range of public and corporate demands, functioning as a “frontline interface” for government services and responses to people’s livelihood issues. Their specific job responsibilities include: answering calls and accurately documenting demands, providing on-the-spot answers to straightforward inquiries or categorizing and forwarding complex issues to relevant authorities, tracking the progress of case handling and urging timely resolution, as well as organizing data and summarizing recurring problems. Professionally, they are required to possess patience, expertise in government policies and service procedures, and the ability to work under pressure. As receivers of public demands, disseminators of policy information, and facilitators of problem-solving, these operators directly influence the “human touch” of government services and the public’s sense of fulfillment from accessing such services. The 12345 Hotline provides 7*24 round-the-clock live service (1).

The predecessor of the 12345 Hotline was the “Mayor’s telephone,” which was established in 1987, when there was only one line and three operators. After more than 30 years, the hotline has expanded to 650 operators and introduced internet- and social media-based service channels (e.g., Weibo) to achieve the whole process of systematic service from consultation to answer, management, and feedback (2). The 12345 hotline can effectively improve the service delivery to citizens, promote law-based administration, innovate social governance, and safeguard the legitimate rights and interests of natural persons, legal persons, and other organizations (1). Due to growing public expectations for the efficiency of hotline services, the Beijing Municipal Government has established a system of “handling complaints as soon as they are received,” which places higher demands on staff (2) efficiency. It is reasonable to imagine that hotline operators experience work pressure, as they are the first contact for hosting an event.

Compassion fatigue refers to negative emotions experienced when delivering certain services or as a result of work-related traumatic experiences. These emotions are similar to the typical symptoms of exhaustion such as anger, depression and frustration (3). According to Stamm (3), compassion fatigue includes two aspects: exhaustion and secondary trauma. For example, helping professionals such as caregivers, social workers, and psychotherapists are prone to compassion fatigue as a result of frequent or repeated witnessing of and listening to the traumatic experiences of their clients (4). Caseload is widely recognized as a contributing factor in compassion fatigue and previous findings have shown a positive correlation between caseload and emotional exhaustion (5).

According to previous surveys, compassion fatigue is common among mental health professionals, with an incidence of approximately 50% (6). Health professionals—particularly those in mental health—are susceptible to job burnout (7). Other research suggests that work environment can affect the degree of job burnout; for example, mental health professionals who work in inpatient settings are more likely to experience burnout than those who work in independent practice settings (8).

Compassion fatigue has many consequences, including emotional and physical problems, high turnover and absenteeism rates, poor-quality services delivered to patients/clients, and a decline in patient/client trust; these consequences can reduce confidence in helping professionals such as hotline operators (9). However, there are few reports investigating psychological stress, job burnout, mental status, and other related factors among 12345 hotline operators. Because people who call for help are always answered with patience, care, respect, and courtesy, the mental status of this group has been overlooked. Given the impact of compassion fatigue on work efficiency and the risk of serious consequences, such as serious societal incidents and negative public opinions of the government, the mental status of this group needs timely attention and effective treatment.

The Minnesota Multiphasic Personality Scale (MMPI) contains multiple validity scales and clinical scales, and is capable of multidimensional assessment of mental health risks. It was originally designed for personality traits and psychopathological assessment in clinical settings. However, after years of development (such as updates in versions like MMPI-2 and MMPI-2-RF), its application has expanded to the field of mental health screening (10). In addition to supporting clinical diagnosis for individuals with mental disorders, the MMPI can also be used to assess the personality traits and mental health status in non-clinical populations (11, 12). Some domestic studies have applied it to stress or mental health screening for specific healthy populations (such as newly recruited employees, college students, and medical staff) (13–15). In this study, the MMPI (16, 566 items) was used to conduct a psychological examination of 12345 hotline operators in City B and explore their personality and psychological characteristics. Through a review of the literature, the 2005 Chinese - localized version of the MMPI test is currently the most widely used version in China. Therefore, this study selects this version of the scale.

## 2 Methods

### 2.1 Participants

A total of 1,010 operators from City B’s 12345 Government Service and Convenience Hotline were invited to complete the Minnesota Multiphasic Personality Inventory (MMPI).(The total number of operators at City B’s 12345 Government Service and Convenience Hotline is 1,010.) The assessment was administered uniformly online during non-working hours—this timing was chosen to minimize fatigue associated with work duties—and was conducted under the guidance and supervision of qualified professionals.

Invalid questionnaires were excluded based on two criteria: (1) incomplete responses with missing items, and (2) obviously perfunctory answers (e.g., consecutive selections of “agree” for all items). After exclusions, 971 valid questionnaires were retained, yielding an overall response rate of 96.1%.

Among the valid participants, 420 were male (43.3%) and 551 were female (56.7%). The sample had a mean age of 25.75 ± 5.51 years, with a range of 18 to 54 years.

### 2.2 Tools

The Minnesota Multiphasic Personality Inventory (MMPI) is one of the most widely used assessments in personality and clinical psychology, as it provides a comprehensive profile of participants’ psychological characteristics (16).

The MMPI comprises 566 items, including 16 repeated items, and is structured into two primary sets of scales:

10 clinical scales: Hypochondriasis, Depression, Hysteria, Psychopathy, Masculinity-Femininity, Paranoia, Psychasthenia, Schizophrenia, Hypomania, and Social Introversion;

3 validity scales: Lie (L) Scale, F Scale (Malingering), and K Scale (Correction).

Additionally, 9 additional scales: Anxiety, Depression, Explicit Anxiety, Ego Strength, Dependence, Dominance, Social Responsibility, Control, and Bias.

The MMPI clinical scales assess dimensions of mental health within participants’ personality structures, while the validity scales measure psychological defense mechanisms and response cooperation (e.g., willingness to answer questions honestly). The supplementary scales, by contrast, are designed to capture participants’ specific emotional states and behavioral patterns (17). Raw scores from individual MMPI subscales are not directly comparable due to differences in item composition and scoring ranges. Thus, raw scores were converted to standardized scores using the formula: T = 50 + 10(X − M)/SD, where:


X=raw score of the subscale,

$$\begin{array}{c} M=\text{mean raw score of the subscale in a normative}\\ \left(\text{normal}\right)\text{reference group}, \end{array}$$

$$\begin{array}{c} SD=\text{standard deviation of the subscale's raw scores }\\ \text{in the normative group}. \end{array}$$

For clinical interpretation, T-scores on each subscale were categorized using established thresholds (18): A T-score of 60 served as the cutoff for abnormality: scores > 60 indicated abnormal functioning, while scores > 70 indicated pathological functioning. Given that extremely high or low T-scores both signal compromised mental status, participants’ overall mental status was stratified into four categories based on subscale T-scores:


Normal:40≤T≤60,

Mildly abnormal:30≤T<40 or 60<T≤70,

Moderately abnormal:20≤T<30 or 70<T≤80,

Severely abnormal:T<20 or T>80.

### 2.3 Statistical analysis

SPSS software (Version 29.0) was used to conduct statistical analyses on the 10 clinical scales, 3 validity scales, and 9 additional scales of MMPI. For operators stratified by mental health status severity, analysis of variance (ANOVA) followed by *post-hoc* tests was applied to examine differences in total MMPI scores and scores across individual factor subscales. Independent samples t-tests were performed to compare MMPI subscale scores between male and female participants. Statistical significance was set at *p* < 0.05; exact values for more stringent significance were reported as “*p* < 0.001” (rather than the abbreviated “*p* <.001”).

### 2.4 Ethical approval and informed consent

This study was approved by the Ethics Committee of Beijing Huilongguan Hospital (Approval Number: 2023-66-Section) and conducted in compliance with relevant ethical guidelines. All participants provided written informed consent prior to enrollment. Before accessing the questionnaire, participants were required to confirm their consent by selecting the “Informed Consent Agreed” option: only those who consented were permitted to proceed to the questionnaire, while participants who selected “Disagree” were automatically redirected out of the survey system and unable to continue.

## 3 Results

### 3.1 Overall MMPI scores

**Figure 1:**
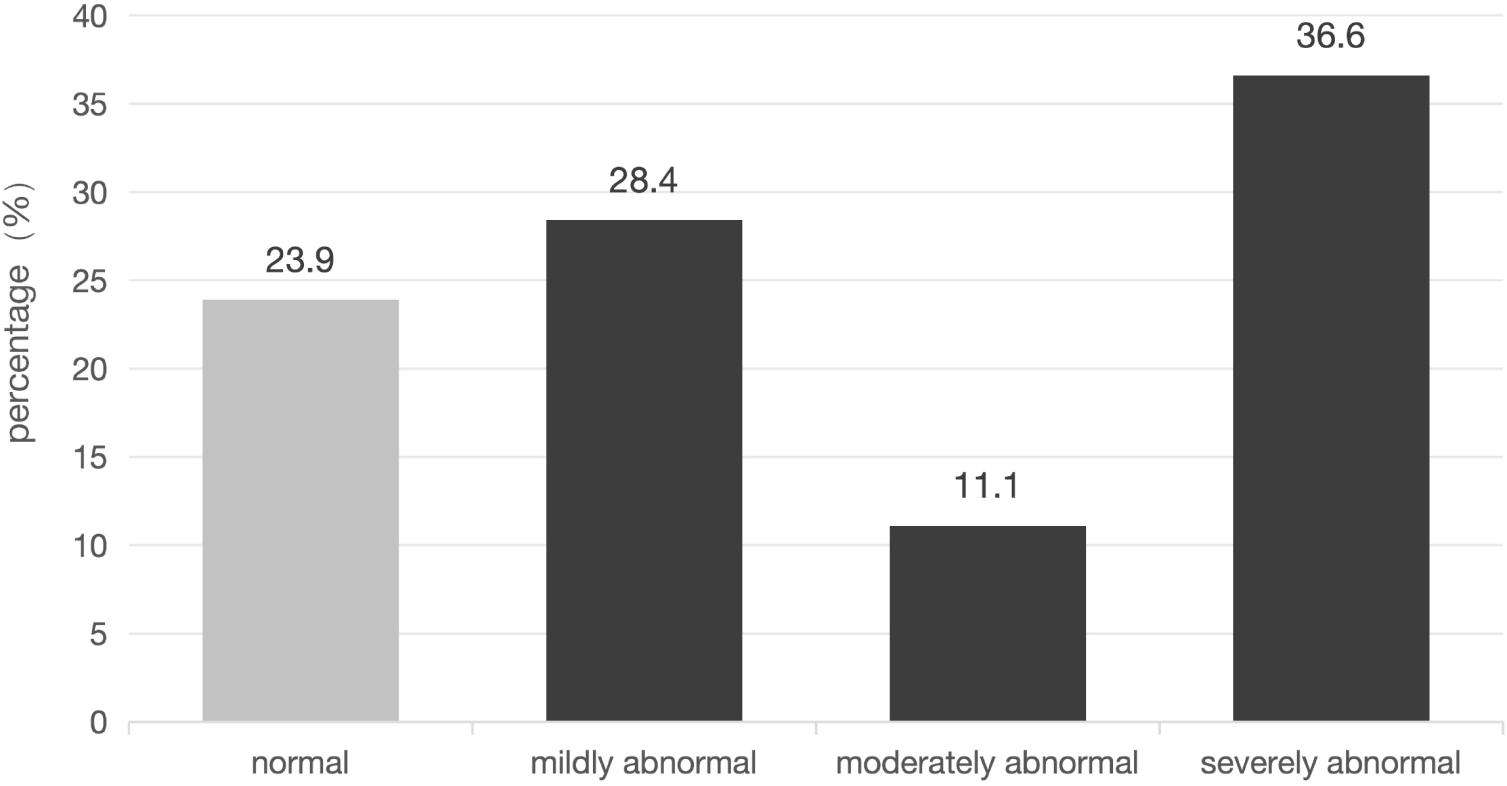
Distribution of mental status abnormality in 12345 hotline operators. (Bar chart showing percentages of normal [23.9%], mildly abnormal [28.4%], moderately abnormal [11.1%], and severely abnormal [36.6%] status; x-axis = mental status category, y-axis = percentage).

As shown in **Figure 1**, 76.1% of 971 participants had abnormal psychological status: 28.4% (n = 276) mild, 11.1% (n = 108) moderate, 36.6% (n = 355) severe; only 23.9% (n = 232) were normal.

**Table 1** shows that mean scores of clinical scales ranged from 46.24 (Masculinity-Femininity) to 57.84 (Hysteria; all within normal range), but variability was high. The highest normal proportions were on Hypomania (68.9%) and Paranoia (68.1%), while the lowest was on Psychasthenia (52.5%).

**Table 1:** MMPI clinical scale scores ( $\bar{X}\pm S$ ) and Normal Proportions [n (%)].

Component scale	Score (n=971)	Normal (40≤x ≤ 60;%)	Mildly abnormal (30≤x<40 & 60<x ≤ 70;%)	Moderately abnormal (20≤x<30 & 70<x ≤ 80;%)	Severely abnormal (x<20/x>80;%)
Hypochondria	56.02 ± 11.43	572(58.9)	303(31.2)	86(8.9)	10(1.0)
Depression	52.07 ± 12.68	550(56.6)	315(32.4)	91(9.4)	15(1.6)
Hysteria	57.84 ± 12.76	528(54.4)	283(29.1)	105(10.8)	55(5.7)
Psychopathy	54.70 ± 11.05	577(59.4)	307(31.6)	73(7.5)	14(1.5)
Masculinity-femininity mininity	46.24 ± 10.57	580(59.7)	328(33.8)	60(6.2)	3(0.3)
Paranoia	52.96 ± 11.02	661(68.1)	217(22.3)	73(7.5)	20(2.1)
Psychasthenia	50.91 ± 13.10	510(52.5)	357(36.8)	98(10.1)	6(0.6)
Schizophrenia	50.82 ± 13.41	518(53.3)	363(37.4)	63(6.5)	27(2.8)
Hypomania	51.26 ± 10.72	669(68.9)	245(25.2)	45(4.6)	12(1.3)
Social introversion	48.18 ± 11.84	612(63.0)	255(26.3)	98(10.1)	6(0.6)

The mean scores of the three validity scales ranged from 52.50 to 54.52, which were all within the normal range; the differences among the participants were still relatively large, with Malingering having the highest normal proportion (56.8%), followed by lying (52.1%) and correction (47.1%) (**Table 2**).

**Table 2:** MMPI validity scale scores ( $\bar{X}\pm S$ ) and Normal Proportions [n (%)].

Component scale	Score (n=971)	Normal (40≤x ≤ 60;%)	Mildly abnormal (30≤x<40 & 60<x ≤ 70;%)	Moderately abnormal (20≤x<30 & 70<x ≤ 80;%)	Severely abnormal (x<20/x>80;%)
Lying	52.50 ± 14.27	506(52.1)	322(33.2)	101(10.4)	42(4.3)
Malingering	53.21 ± 14.32	552(56.8)	286(29.5)	63(6.5)	70(7.2)
Correction	54.52 ± 15.15	457(47.1)	311(32.0)	153(15.8)	50(5.1)

For additional scales, only the Depression Factor had a mean score exceeding 60 (60.35), and its normal proportion was the lowest (45.2%); Ego Strength had the highest normal proportion (74.9%) (**Table 3**).

**Table 3:** MMPI additional scale scores ( $\bar{X}\pm S$ ) and Normal Proportions [n (%)].

Component scale	Score (n=971)	Normal (40≤x ≤ 60;%)	Mildly abnormal (30≤x<40 & 60<x ≤ 70;%)	Moderately abnormal (20≤x<30 & 70<x ≤ 80;%)	Severely abnormal (x<20/x>80;%)
Anxiety factor	45.53 ± 8.96	614(63.2)	348(35.9)	9(0.9)	0(0)
Depression factor	60.35 ± 12.37	439(45.2)	318(32.7)	153(15.8)	61(6.3)
Explicit anxiety	51.09 ± 10.14	658(67.8)	276(28.4)	33(3.4)	4(0.4)
Ego strength	47.93 ± 9.01	727(74.9)	218(22.4)	26(2.7)	0(0)
Dependence	47.54 ± 12.00	523(53.9)	371(38.2)	77(7.9)	0(0)
Dominance	51.09 ± 10.14	607(62.5)	327(33.7)	36(3.7)	1(0.1)
Social responsibility	44.13 ± 8.76	678(69.8)	228(23.5)	58(6.0)	7(0.7)
Control	51.34 ± 9.86	668(68.8)	268(27.6)	35(3.6)	0(0)
Bias	46.90 ± 11.53	563(58.0)	334(34.4)	69(7.1)	5(0.5)

### 3.2 MMPI differences by mental status

One-way ANOVA showed significant differences in all MMPI scales (except Depression Factor) across mental status groups (all *p* < 0.001; **Table 4**). *Post-hoc* tests revealed:

**Table 4:** Scale scores of the MMPI of 12345 hotline operators with different degrees of mental status ( $\bar{X}\pm S$ ).

Component scale	Normal (*n* = 232)	Mildly abnormal (*n* = 276)	Moderately abnormal (*n* = 108)	Severely abnormal (*n* = 355)	*F*	*P*	*Eta square*
Hypochondria	44.32 ± 6.24	52.78 ± 7.18	59.90 ± 8.23	65.01 ± 9.40	339.51	<0.001	0.51
Depression	41.17 ± 8.22	49.93 ± 9.14	54.42 ± 10.48	60.15 ± 12.25	163.98	<0.001	0.34
Hysteria	51.57 ± 7.14	55.44 ± 9.66	58.82 ± 11.35	63.51 ± 15.44	52.77	<0.001	0.14
Psychopathy	45.45 ± 7.26	51.68 ± 8.45	56.71 ± 8.69	62.46 ± 9.85	191.85	<0.001	0.37
Masculinity -femininity mininity	44.61 ± 9.45	44.63 ± 11.00	45.94 ± 9.50	48.65 ± 10.83	10.42	<0.001	0.03
Paranoia	44.82 ± 5.79	48.01 ± 7.24	54.28 ± 7.72	61.71 ± 10.59	233.96	<0.001	0.42
Psychasthenia	39.23 ± 6.89	47.90 ± 8.89	56.01 ± 10.80	59.33 ± 12.93	191.94	<0.001	0.37
Schizophrenia	38.68 ± 6.20	46.06 ± 8.08	54.29 ± 9.15	61.40 ± 12.71	282.54	<0.001	0.47
Hypomania	47.41 ± 7.98	48.44 ± 8.46	53.85 ± 10.07	55.17 ± 12.40	38.14	<0.001	0.11
Social introversion	38.73 ± 8.70	47.13 ± 10.75	51.09 ± 8.86	54.28 ± 11.05	112.38	<0.001	0.26
Lying	56.51 ± 13.12	52.04 ± 12.48	46.76 ± 12.09	51.98 ± 16.11	12.62	<0.001	0.04
Malingering	41.48 ± 5.74	46.91 ± 7.54	54.44 ± 9.19	65.39 ± 14.14	299.65	<0.001	0.48
Correction	60.69 ± 11.26	54.80 ± 12.96	49.61 ± 13.97	51.77 ± 17.79	21.86	<0.001	0.06
Anxiety factor	39.05 ± 4.87	43.83 ± 6.57	48.37 ± 7.70	50.23 ± 9.94	105.84	<0.001	0.25
Depression factor	*59.21 ± 8.51*	*60.85 ± 10.32*	*59.75 ± 11.43*	*60.88 ± 15.76*	*1.11*	*0.35*	
Explicit anxiety	41.47 ± 5.52	49.56 ± 7.38	56.09 ± 8.41	58.56 ± 9.57	230.37	<0.001	0.42
Ego strength	57.02 ± 4.49	49.66 ± 5.87	44.59 ± 6.59	41.66 ± 8.39	259.38	<0.001	0.45
Dependence	38.40 ± 7.47	45.69 ± 9.12	52.37 ± 10.04	53.49 ± 12.76	109.58	<0.001	0.25
Dominance	56.27 ± 6.96	52.68 ± 8.32	49.44 ± 9.28	46.96 ± 11.52	49.50	<0.001	0.13
Social responsibility	50.09 ± 6.73	45.99 ± 6.98	41.18 ± 7.52	39.68 ± 8.78	96.75	<0.001	0.23
Control	45.78 ± 9.04	51.04 ± 8.66	56.54 ± 9.62	53.63 ± 9.65	46.89	<0.001	0.13
Bias	39.68 ± 7.84	44.37 ± 8.73	51.45 ± 9.60	52.21 ± 12.78	81.87	<0.001	0.20

Moderate-to-severe abnormal groups had significantly higher scores on Hypomania (clinical), Correction (validity), Dependence, Social Responsibility, and Bias (additional) than normal-to-mild groups (all *p* < 0.001);Severely abnormal group had significantly higher Masculinity-Femininity scores than the other three groups (vs. normal/mild: *p* < 0.001; vs. moderate: *p* < 0.05).

### 3.3 Gender differences in MMPI results

An independent samples t-tests was conducted to examine gender differences MMPI scores among 12345 hotline operators, which showed significant differences in the 10 clinical scales, 3 validity scales, and 9 additional scales for different genders.

Male respondents scored significantly higher than females on four clinical subscales: masculinity-femininity, psychasthenia, schizophrenia, and hypomania (*p* < 0.05) (**Table 5**).

**Table 5:** Comparison of MMPI clinical scale scores between male and female 12345 hotline operators ( $\bar{X}\pm S$ ).

Component scale	Male(*n* = 420)	Female(*n* = 551)	*t*	*P*	Cohen’s *d* value
Hypochondria	56.84 ± 11.48	55.40 ± 11.36	1.94	0.053	
Depression	51.43 ± 12.03	52.57 ± 13.15	-1.39	0.166	
Hysteria	57.71 ± 12.84	57.95 ± 12.70	-0.29	0.773	
Psychopathy	55.02 ± 11.46	54.44 ± 10.73	0.81	0.419	
Masculinity-femininity	51.46 ± 9.20	42.26 ± 9.80	14.88	0.000	0.97
Paranoia	53.54 ± 12.07	52.51 ± 10.13	1.42	0.157	
Psychasthenia	51.98 ± 13.17	50.09 ± 12.99	2.22	0.026	0.14
Schizophrenia	52.29 ± 14.00	49.70 ± 12.85	2.95	0.003	0.19
Hypomania	52.30 ± 11.56	50.47 ± 9.97	2.59	0.010	0.17
Social introversion	47.99 ± 11.34	48.32 ± 12.22	-0.44	0.662	

On the validity scale, the scores of females on the correction subscale were significantly higher than those of males (*p* < 0.01) (**Table 6**).

**Table 6:** Comparison of MMPI validity scale scores between male and female 12345 hotline operators ( $\bar{X}\pm S$ ).

Component scale	Male(*n* = 420)	Female(*n* = 551)	*t*	*P*	Cohen’s *d* value
Lying	52.10 ± 14.15	52.80 ± 14.37	-0.75	0.453	
Malingering	53.72 ± 13.93	52.82 ± 14.60	0.97	0.333	
Correction	52.62 ± 14.93	55.98 ± 15.17	-3.45	0.001	-0.22

**Figure 2:**
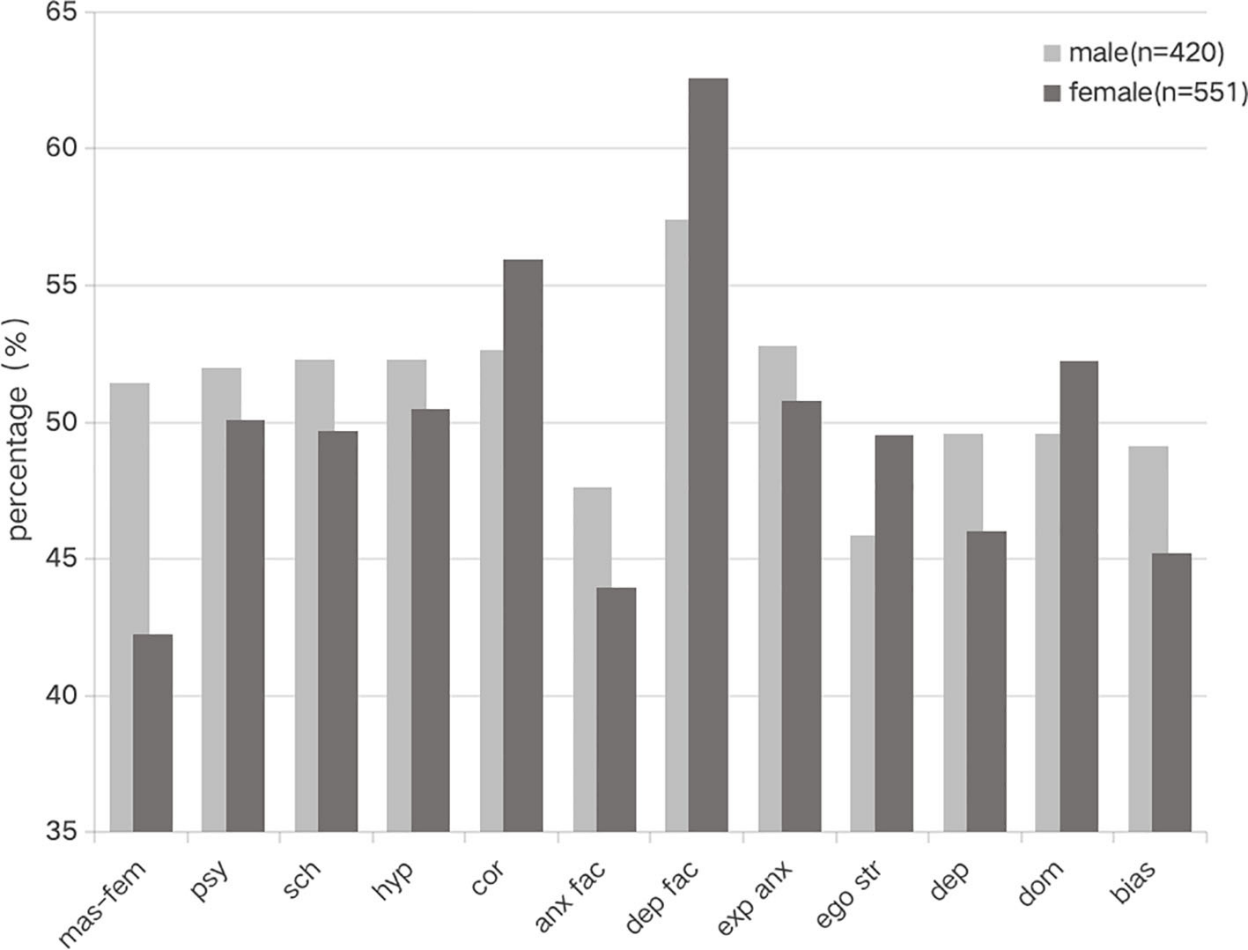
Gender differences in MMPI scores among 12345 hotline operators.

In the additional scales, males scored on the four subscales of anxiety, explicit anxiety, dependence, and bias were higher than those of females. By contrast, their scores on the three subscales of depression factor, ego strength, and dominance were lower than those of females; the differences were all statistically significant (*p* < 0.01) (**Table 7**). The gender differences in MMPI results are presented in **Figure 2**.

**Table 7:** Comparison of MMPI additional scale scores between male and female 12345 hotline operators ( $\bar{X}\pm S$ ).

Component scale	Male(*n* = 420)	Female(*n* = 551)	*t*	*P*	Cohen’s *d* value
Anxiety factor	47.61 ± 8.52	43.94 ± 8.98	6.46	0.000	0.42
Depression factor	57.40 ± 11.91	62.60 ± 12.24	-6.63	0.000	-0.43
Explicit anxiety	52.80 ± 10.68	50.76 ± 10.22	3.03	0.003	0.20
Ego strength	45.85 ± 8.70	49.51 ± 8.94	-6.41	0.000	-0.42
Dependence	49.57 ± 11.92	46.00 ± 11.83	4.65	0.000	0.30
Dominance	49.59 ± 10.12	52.23 ± 10.02	-4.06	0.000	-0.26
Social responsibility	44.35 ± 8.39	43.96 ± 9.03	0.68	0.497	
Control	51.99 ± 10.18	50.84 ± 9.59	1.80	0.073	
Bias	49.14 ± 11.65	45.20 ± 11.15	5.36	0.000	0.35

## 4 Discussion

### 4.1 Key findings

Previous studies have primarily focused on two areas: the mental health status of individuals seeking support through hotlines (19–24) and the role of hotline services in improving callers’ mental health (20–22, 25–27). However, limited research has examined either the mental status of hotline operators themselves or the impact of hotline work on operators’ mental health.

Recent research notes that during major global public health events—such as the COVID-19 pandemic—support hotlines faced numerous challenges, including a surge in consultation demand and increasing diversity in callers’ concerns (28, 29). Building on this context, the present study found that the average mental status of 12345 hotline operators was suboptimal, with an abnormality rate of 76.1%. These operators interact directly with members of the public, and their work requires responding to a wide range of diverse issues while ensuring timely information transmission (i.e., “uploading and delivering” as referenced in local administrative contexts). Such demands necessitate frequent emotional regulation and generate heightened emotional strain, thereby exerting a more pronounced impact on their mental health.

An interview study of National Health Service (NHS) call handlers in the UK documented the direct adverse impacts of workload overload: high call volumes, prolonged call durations, and complex, challenging inquiries were found to directly contribute to emotional exhaustion, physical and mental fatigue, and even feelings of helplessness and frustration (Oriental Information (30). Maslach’s research highlights that work overload is one of the key contributing factors to burnout (31). In the present study, the high rate of abnormal MMPI results likely indicates that 12345 hotline operators are experiencing substantial emotional exhaustion and occupational stress—highlighting the need to proactively address the potential risk of job burnout in this population.

Notably, for the MMPI additional scales, fewer than 50% of participants scored within the broadly normal range on the Depression scale. Furthermore, the Depression scale was the only one across all measured scales where the mean score exceeded the threshold for non-normal functioning (i.e., T > 60; see Method section for threshold definitions). Clinically, such elevated Depression scale scores manifest as a range of symptoms, including physical discomfort, poor environmental adaptability, and reduced work and learning efficiency (16).

Results from the one-way ANOVA revealed that operators with moderate-to-severe mental status abnormalities differed significantly from those with normal-to-mild abnormalities across five MMPI dimensions: Hypomania, Correction (K Scale), Dependency, Social Responsibility, and Bias. Specifically, operators with moderate-to-severe abnormalities exhibited more pronounced psychological difficulties compared to their peers with normal or mild mental status concerns.

Consistent with prior research, dependent personality tendencies have been identified as predisposing and risk factors for depression (32). Additionally, lower scores on the Social Responsibility scale indicate a reduced willingness to take on social responsibilities and endorse social values or behavioral norms—further reflecting the psychological strain experienced by this group.

Notably, operators with severe mental status abnormalities showed significant differences in Masculinity-Femininity (Mf) subscale scores relative to the other three mental status groups (normal, mild, moderate). This finding suggests that operators with severe abnormalities may harbor doubts about their personal capabilities, as the Mf subscale indirectly reflects self-perceptions of competence and identity coherence in occupational contexts.

Relevant to the Bias dimension, an intervention study focused on recognizing and managing implicit bias found that participants reported greater self-reported confidence and comfort across all assessment items following the intervention (33). This supports the potential value of targeted interventions to address psychological vulnerabilities (e.g., bias-related distress) among 12345 hotline operators.

### 4.2 Gender differences

Regarding gender differences in stress- and burnout-related factors, findings across relevant studies exhibit slight inconsistencies—though most highlight notable gender disparities, with a few exceptions.

For instance, Lyubarova et al. (34) found that female physicians experience substantially higher burnout than their male counterparts, with this disparity attributed to multiple contributing factors. Similarly, Briggs et al. (35) noted that overall burnout rates (75.6%) among their sample exceeded historical norms, and female students specifically reported higher burnout levels than male students. Among surgical faculty, Lu et al. (36) further identified gender differences that drive both career dissatisfaction and burnout. Rotenstein et al. (37) reinforced this trend by demonstrating that female physician faculty have higher burnout rates and lower levels of professional fulfillment compared to male faculty. Among academic populations, Redondo-Flórez et al. (38) reported that female professors exhibit higher levels of burnout, perceived stress, emotional exhaustion, and neuroticism than their male peers.

Notably, some studies have uncovered more nuanced or contrasting patterns. Alenezi et al. (39) conducted an online cross-sectional survey on U.S. nurse leaders during the COVID-19 pandemic and found gender-specific burnout profiles: females had significantly higher personal burnout, while males reported significantly higher client-related burnout—underscoring the need to consider context-specific gender dynamics when examining burnout. In a separate study on VA primary care providers (PCPs), Apaydin et al. (40) observed that nearly half of the sample (47.6%) reported burnout, but no significant gender difference in overall burnout rates was detected.

In the present study, male operators scored higher on the Masculinity-Femininity (Mf) scale, a finding that reflects more assertive behavioral tendencies (41). Additionally, higher Hypomania scale scores among male operators may indicate greater adaptability to high-pressure tasks (3), whereas female operators’ higher Ego Strength scores suggest stronger resilience to emotional stress (42). The findings suggest that male and female hotline operators each exhibit distinct strengths relevant to hotline service delivery. These gender-specific patterns highlight the need for tailored interventions—for example, prioritizing stress management support for males and targeted emotional support for females.

These observed gender differences align with neurofunctional research: female brains demonstrate greater sensitivity to emotional stimuli, while male brains exhibit stronger logical reasoning abilities (41). This neurobiological distinction may correspond to the distinct demands of hotline work—for instance, requiring more emotional regulation from females and more problem-solving from males—further contextualizing the gender-specific mental health patterns identified in this study.

Notably, this study did not observe higher burnout rates among female operators, a result that contrasts with findings from Mangoulia et al. (43). One potential explanation is that female operators’ higher scores on the Dominance scale may enhance their capacity to cope with job demands (44), offsetting the burnout risk typically associated with gender in prior research.

### 4.3 Practical implications and recommendations

A study of trauma-exposed workers found that greater social support was associated with lower levels of compassion fatigue (42). Drawing on this finding, the following recommendations are proposed to improve the mental status of 12345 hotline operators:

1. Organizational support: Relevant administrative departments should prioritize the provision of practical, targeted support services. Specifically, they could implement staff stress-reduction training programs to alleviate work-related pressure among hotline operators and enhance their mental well-being (45). Additionally, team-building initiatives and one-on-one check-in sessions centered on operators’ subjective experiences and psychological needs should be organized (46), with consistent, timely attention paid to monitoring the mental status of this workforce.
2. Supervisor training: Supervisors should receive specialized training to enable early identification of burnout indicators, such as increased absenteeism and declining response efficiency (7).
3. Pre-employment screening: The MMPI should be integrated into the recruitment process to identify candidates with high ego strength—an attribute associated with stronger job adaptability (45).

### 4.4 Limitations

This study is subject to several limitations, as outlined below:

#### 4.4.1 COVID-19-related confounding bias

Data collection occurred during the late stage of the COVID-19 pandemic. The pandemic-induced livelihood challenges exacerbated unpredictable social tensions, which in turn imposed unprecedented occupational stress on 12345 hotline operators. Specifically, operators were required to mobilize additional psychological and emotional resources to address unforeseen caller needs and respond to heightened emotional distress among a larger volume of clients—factors that likely influenced their mental status. Such elevated work intensity and fatigue may have introduced confounding effects on the questionnaire results. Future research should seek to control for these extraneous variables.

#### 4.4.2 Self-report bias

The MMPI is a self-administered assessment tool, and its reliance on participants’ subjective evaluations may introduce response bias. Specifically, social desirability bias—where participants tend to present themselves in a socially favorable light—may have led to underreporting of severe psychological symptoms (10).

#### 4.4.3 Selection bias

There may have been a self-selection bias, whereby operators experiencing higher levels of stress were more likely to complete the questionnaire. This potential overrepresentation of stressed individuals could have led to an overestimation of the prevalence of mental status abnormalities.

#### 4.4.4 Cross-sectional study design

Given the cross-sectional nature of this study, we were unable to establish causal relationships between job demands and operators’ mental status. Future research could adopt longitudinal follow-up designs or interventional studies to identify effective, sustainable strategies for improving the psychological well-being of hotline operators.

#### 4.4.5 Limitations of assessment tools

The MMPI lacks occupation-specific dimensions (e.g., burnout), which may have restricted the comprehensiveness of our mental status evaluations. To address this gap, future studies could integrate additional validated instruments—such as the Maslach Burnout Inventory (MBI) or the Professional Quality of Life Scale (ProQOL; 3)—to more accurately capture constructs like compassion fatigue and burnout in this occupational group.

## 5 Conclusion

In summary, the mental status of 12345 hotline operators was found to be suboptimal. Specifically, operators with moderate-to-severe mental health abnormalities exhibited more pronounced psychological issues in domains including hypomania, correction, dependency, social responsibility, and bias. Additionally, gender differences were identified across multiple dimensions of mental status. Train supervisors to recognize early signs of burnout. Training supervisors to recognize the early signs of burnout is not only important but also meaningful, as it helps ensure the smooth operation of 12345 Government Service Hotline services.

## Data Availability

The original contributions presented in the study are included in the article/supplementary material. Further inquiries can be directed to the corresponding author.
